# Nutritional Assessment of Plant-Based Meat Products Available on Hong Kong Market: A Cross-Sectional Survey

**DOI:** 10.3390/nu15173684

**Published:** 2023-08-22

**Authors:** Qile Zhang, Yilin Liu, Chufeng He, Ruiwen Zhu, Minghui Li, Hon-Ming Lam, Wing-Tak Wong

**Affiliations:** 1 School of Life Sciences, Faculty of Science, The Chinese University of Hong Kong, Hong Kong, China; zhangqile@link.cuhk.edu.hk (Q.Z.); 1155134757@link.cuhk.edu.hk (C.H.); 1155138939@link.cuhk.edu.hk (R.Z.); 2 The Jockey Club School of Public Health and Primary Care, Faculty of Medicine, The Chinese University of Hong Kong, Hong Kong, China; 1155189592@link.cuhk.edu.hk; 3 School of Pharmacy, University College London, London WC1N 1AX, UK; zczqmli@ucl.ac.uk; 4 State Key Laboratory of Agrobiotechnology, The Chinese University of Hong Kong, Hong Kong, China

**Keywords:** plant-based meat, nutrient profile, salt, sugar

## Abstract

Background: Plant-based meat (PBM) takes up ever-increasing market shares and draws great attention from both customers and retailers these days. However, little is known about the nutritional quality of PBM products. Objective: This study intended to profile and evaluate the overview nutrition of PBM with equivalent meat products on the Hong Kong market. Methods: We conducted a cross-sectional survey of 274 PBM and 151 meat products from 27 different brands on the Hong Kong market in October 2022. The nutritional differences between PBM and meat products were assessed using analysis of covariance (ANCOVA) and two independent sample t-test. The nutritional quality of PBMs was evaluated according to nutrient reference value, front-of-package (FoP) criteria and nutritional score. Results: PBM had relatively lower energy density, total fat, saturated fat, protein, and salt compared to meat. According to the FoP criteria, 91.36%, 17.88%, and 99.34% of PBMs were labeled as medium to high in fat, salt, and sugar, respectively. Through ingredient analysis of 81 PBM products, soy and canola were the main source of protein and fat. Conclusions: PBM products have a roughly better nutrient quality compared to muscle-based meat, though there is still potential for further refinement in terms of production, consumption, and regulation.

## 1. Introduction

The global healthy diet cannot only comprise animal-source meat and milk. Plant-rich fruits, vegetables, whole grains, and plant-source meats are promoted by public health practitioners and nutritionists nowadays [1]. The market demand for plant-based meat has substantially increased. It is estimated that the global plant-based meat market value is expected to reach USD 30.92 billion by 2026 from USD 10.10 billion in 2018, growing at a compound annual growth rate of 14.8% [2]. The huge market value has gained the investment of several restaurant giants. For example, Burger King has teamed up with plant-based meat industry giant IMPOSSIBLE MEAT to launch its latest product that has hit multiple markets [3].

Meat analogues developed by the food industry include alternatives to beef, pork, lamb, seafood, etc. [4]. The meat alternatives simulate not only meat types, but also forms of meat-made products, including plant-based burgers, plant-based fish fingers, plant-based sausages, etc. As new-generation products, PBMs are developing to reach the same appearance, texture, taste, and flavor as meat through alternative content sources [5,6]. For example, traditional protein sources are soy and wheat. However, some food factories turn to texturized vegetable proteins as upgradation.

Many previous studies have pointed out that plant-based meat has not only an obviously positive impact on health, but also environmental advantages in comparison to meat [1,7,8]. For health, some studies have demonstrated that plant-based products and diets can mitigate the risk of cardiovascular disease by detecting the biomarker trimethylamine-N-oxide (TMAO) in blood and urine [9,10]. For environmental impact, ingredients such as grains, legumes, nuts, seeds, etc., used in PBM products produce fewer emissions of greenhouse gases than animal products [11]. However, not all plant-based foods are good for your health. Due to the fact that a great number of PBMs are ultra-processed foods (UPFs) [12], the benefits of plant-based dietary have been modified [13].

Furthermore, PBM acceptance and consumption are impacted by several factors. Firstly, food health is a critical factor to customer’s preferences. Although much attention has been drawn to plant-based meat, citizens still doubt these novel products due to nutrition and safety concerns. A survey on Beijing customers demonstrates that people will prefer PBMs when more nutrition information is provided [14]. In addition, naturalness is another key for people’s choice of PBMs, especially the technology used and the ingredients of the final products [15]. A recent study revealed that people prefer PBMs with similar texture and appearance to traditional meat products [16], indicating that used ingredients of PBMs are also dependent on sensory attributes and consumer acceptance. Moreover, characteristics of PBM consumers also affect PBM acceptance. Females, millennials, and high-income workers report dramatically higher PBMs intake [17]. Conservative consumers are not willing to try PBMs, revealing that political ideology plays a role in PBMs consumption [18]. Notably, it has been reported that the price of plant-based beef is approximately twice that of authentic beef. A mountain of work is required if people try to raise PBM intake by lowering its price [19].

So far, several countries including the UK, Sweden, and the USA have conducted nutrition-related cross-sectional studies of local PBM markets. Overall, PBMs have more fiber and lower saturated fat, total fat, and energy density than meat [20,21]. However, due to different brands available in the local market and different criteria, the proportion of products labelled as healthy varies from country to country. The nutritional quality of PBMs varies between product categories. For example, compared to other PBM categories, nugget and fillet analogues are relatively healthier [20]. There are also a few studies that conduct analysis on a certain specified category. According to a study on plant-based ground beef alternative products available in the USA, most of the products contained less protein, zinc, and vitamin B12 than ground beef [22]. Cole, et al. [23] found alternative beef burgers contain more vitamin D, calcium, and iron than beef burgers.

Diverse PBM products are available on the Hong Kong market, including both imported brands such as “GARDEIN” and “BellRing” and local brands such as ‘Soo Good Vegan’. However, little was known about the local nutritional quality of PBMs. In addition, given that there are many vegetarians along with great concerns about dietary health in Hong Kong, there is a need to figure out the nutrition profile of PBM.

Our study intends to compare the nutritional content of PBM products retailed in Hong Kong with the corresponding meat products using the local “front of package” labeling standards. The second objective of this study is to profile and evaluate the overview nutrition of PBM available in Hong Kong markets. The third goal is to further analyze the major nutrient source substances in the PBM product ingredient list to provide suggestions for the regulation, development, and consumption of PBMs from a nutritional point of view.

## 2. Materials and Methods

### 2.1. Study Design

We carried out a cross-sectional survey of PBM products in November 2022.

PBM products were selected according to the following inclusion criteria:

- Products made of fungal or plant-based ingredients but are designed to mimic the taste, texture, and full consumer experience of meat.
- Semi-finished PBM products or instant packaged PBM products.
- PBM products that were lightly breaded, fried, or had flavor sauce.
- PBM products which are designed to imitate a certain meat product (e.g., chicken tender, sausage).
- The same formulation products in different package sizes should be included only once. The same type of products but in different flavors should be included.

Meat products are selected in correspondence to PBM products. We included processed and unprocessed products made of beef, pork, poultry, and seafood.

### 2.2. Data Collection

#### 2.2.1. Product Selection

We select PBMs and meats products from Wellcome, ParknShop, 7-Eleven, Circle K, and HKTV Mall. Wellcome and ParknShop dominated the supermarket category with over 90 percent market share combined [24]. 7-Eleven and Circle K hold more than 99% of profiles of convenience stores in Hong Kong [24]. HKTV Mall had the most web visits in e-commerce platforms [25]. These five chains jointly hold more than 90% of the grocery market share in Hong Kong. We also collected PBMs data from Green Common, Whole Foods, and Planet Organic, as these chains are known for offering a wide range of PBM products.

We collected data from 2–3 large spots of each main retailer in Hong Kong to ensure accuracy of sample inclusion.

We retrieved nutrition information from food labels on products and transformed per serving data into per 100 g. For nutrient content labeled as “<0.1”, it was replaced by 0.1.

#### 2.2.2. Product Categorization

**Figure 1:**
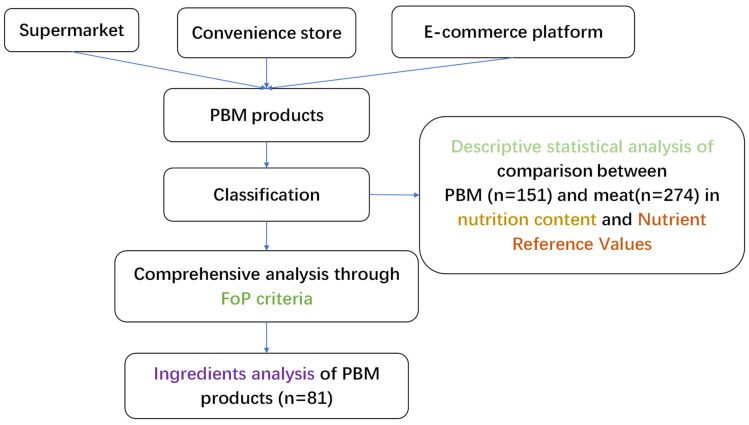
The selection process used for the inclusion of PBM and meat products in the nutrient assessment.

PBMs stimulate the taste and types of meat products. Different ingredients are added to achieve an “analogue” effect, which may cause variation in nutrients. To have a more comprehensive and specific understanding of nutritional quality of PBM products, we processed PBM and corresponding meat data in two classification ways. Classification Ⅰ divides PBM data into 4 categories from what kind of animal meat analogue they are (plant-based pork, plant-based beef, plant-based seafood, and plant-based poultry). Classification Ⅱ divides PBM data into 7 categories according to different processed product types (burger, sausage, mince, jerky, meatball, plain meat analogue, and breaded meat analogue). Meat products are categorized in correspondence to the two classifications of PBM. Table 1 (a), (b) show the categories and corresponding description. The framework (Figure 1) shows the inclusion of PBM and meat products in the classification and analysis process.

### 2.3. Statistical Analysis of Nutrient Content

We carried out the overall nutritional comparison (i.e., total fat, saturated fat, protein, carbohydrate, sugar, salt, and energy density) for PBM and meat products first. The weight average for these macronutrients was calculated under two classifications with weights for each type of PBM products applying to meat products. Analysis of covariance (ANCOVA) was used to assess the significance of net difference after controlling the types of products.

Furthermore, specific analysis was conducted with descriptive statistics (mean, SD, range) for total fat, saturated fat, protein, carbohydrate, sugar, and salt (g/100 g) and for energy density (kcal/100 g) for all PBMs and their corresponding meat categories, and *p*-value derived from two-sample t-testing for the assessment of significant difference of each class. For visual presentation, a horizontal bar chart was displayed with mean nutrient content of meat products as the baseline, showing percentage difference of corresponding PBM products.

### 2.4. Nutrition Assessment

#### 2.4.1. Nutrient Reference Values in Relation to Chinese Dietary Reference Intakes (CDRI)

The percentage of Nutrient Reference Values (NRV) to A for 100 g of PBMAs and meat references was calculated [26]. The calculation was based on the percentage of daily reference value (energy), recommended intake (fat, protein, carbohydrates, salt) and maximum recommended intake (saturated fat) according to Table 2.

The calculation formula [27] which is designed by the Chinese Centre for Disease Control and Prevention (CCDCP) is:

X/NRV × 100% = Y %

X = the amount of a nutrient in the food

NRV = Nutrient reference value for that nutrient

Y % = Calculation result

#### 2.4.2. Evaluation Using Front-of-Pack (FoP) Criteria

We referenced the standard from Table 3 to assess the content of total fat, saturated fat, sugar, and salt in each plant-based meat product [27]. The FoP standard is also complying with the Reduction of Dietary Sodium and Sugar in Hong Kong [28], which is used to assess the content of sugar (≤0.5 g/100 g as zero, ≤5 g/100 g as low) and salt (≤0.0125 g/100 g as zero, ≤0.3 g/100 g as low).

### 2.5. Ingredients Analysis

As PBMs are manufactured products, we collected information on the ingredient lists of PBM products and analyzed their thickeners, which are claimed on food ingredient list, and main sources of protein and oils.

## 3. Results

Nutrient data were collected from 151 PBM products which are from 27 different brands (Appendix A) and 274 meat products. The data were classified in two ways for nutrient content analysis. Under classification Ⅰ, 145 PBM products (excluded six uncategorized PBM products) and 268 meat products (excluded six uncategorized meat products) were involved according to different meat types (i.e., pork, beef, poultry, and seafood). Under classification Ⅱ, 148 PBM products (excluded n = 3) and 274 meat products were involved.

### 3.1. Nutrients Comparison of All the PBMs and Meats

#### 3.1.1. Overall Comparison

**Figure 2:**
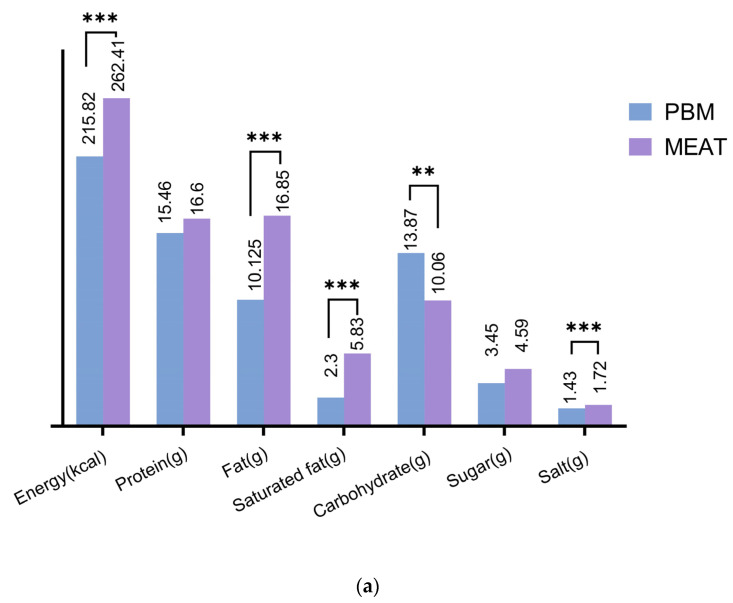

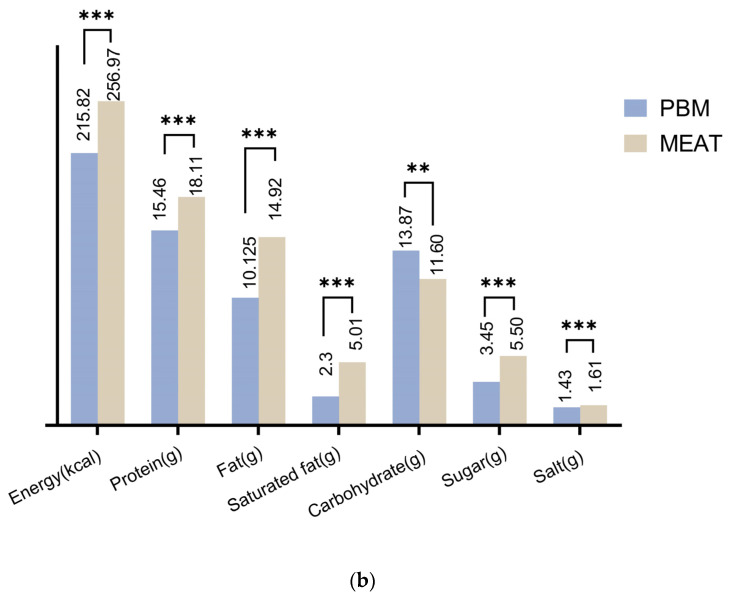
(**a**) Nutrition Contribution Comparison between meat and PBM according to classification I (weighted mean). Asterisks indicate significant differences between all PBMs and all meat references controlling for types of products. (** *p* < 0.01; *** *p* < 0.001). (**b**) Nutrition Contribution Comparison between meat and PBM according to classification II (weighted mean). Asterisks indicate significant differences between all PBMs and all meat references controlling for types of products. (** *p* < 0.01; *** *p* < 0.001).

Figure 2a,b demonstrate the overall comparison (weighted mean) provided by 100 g plant-based meat and meat references under two classification methods, respectively. Weight based on the proportion of product attributing to different PBM categories was applied to corresponding meat for generating weighted average values, and ANCOVA was used to judge statistical difference of PBM and meat after adjusting for type of products. Combining two classifications, the results (Figure 2a,b) indicated that although the weights of the product types under the two classifications differ, resulting in variations in the adjusted mean, the underlying distribution remains essentially similar. The results of ANCOVA revealed that not all disparities attained statistical significance, particularly concerning classification I, where no significant differences were observed between carbohydrates and sugars (15.46 g/100 g versus 16.6 g/100 g, *p* > 0.05). In contrast, in classification Ⅱ, the variations of all nutrients exhibited statistical significance. Specifically, the macronutrients’ content of PBM was significantly lower than that of meat products, except for carbohydrates, which demonstrated contrary results (13.87 g/100 g versus 11.60 g/100 g, *p* < 0.0001).

#### 3.1.2. Subgroup Comparison

**Figure 3:**
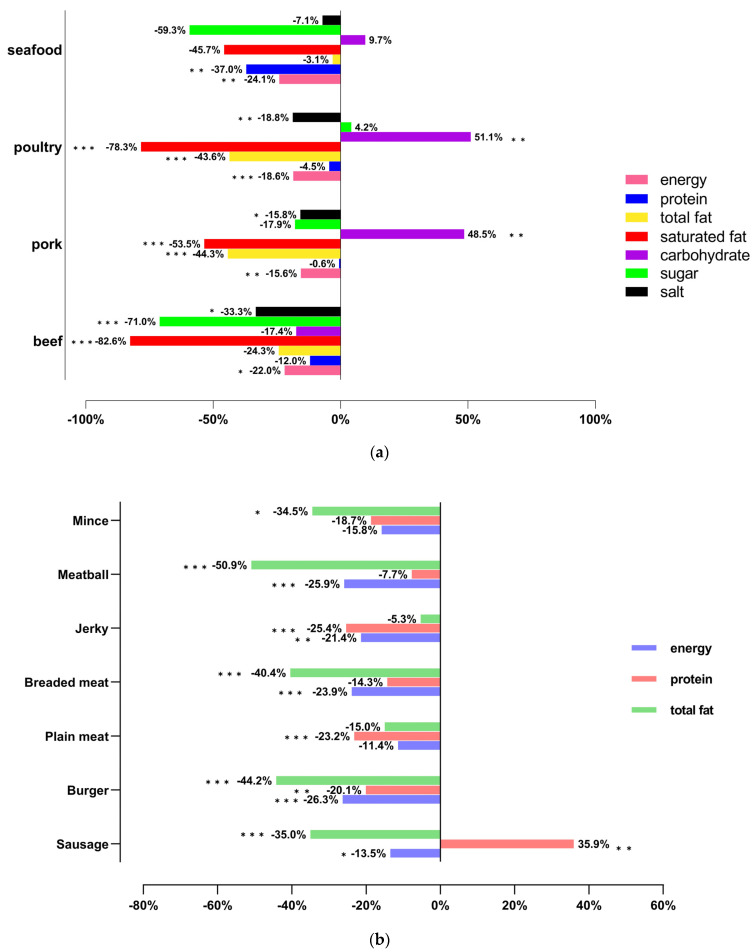
(**a**) Mean energy density (kcal/100 g) and nutrient content (g/100 g) ± SD and range of meat and PBM categories in classification Ⅰ (meat as the reference baseline). (**b**) Mean energy density (kcal/100 g) and nutrient content (g/100 g) ± SD and range of meat and PBM categories in classification Ⅱ (meat as the reference baseline). (* *p* < 0.05; ** *p* < 0.01; *** *p *< 0.001).

Figure 3a,b depicted the disparity in content by considering meat as the reference baseline and calculated the discrepancies as the percentage of corresponding plant-based meat. The specific means (sd) of each category, along with corresponding *p*-value obtained from two independent sample *t*-test, are presented in Appendix A.

- Energy density: The energy density of 100 g plant-based meat was statistically lower than that of equivalent meat products in all the categories under both classifications.
- Total fat: Total fat was over 40% lower in both pork and poultry analogue than corresponding meat catalogue (19.2 ± 8.8 vs. 10.7 ± 5.5, *p* < 0.0001; 15.6 ± 6.4 vs. 8.8 ± 4.3, *p* < 0.0001) according to classification Ⅰ. For classification Ⅱ, the circumstance was similar except for jerky and plain meat with its plant-based analogue (no significant difference).
- Saturated fat: Saturated fat was significantly lower in all PBM categories and classifications (except plant-based seafood and seafood). For example, the average saturated fat content in beef products was over five times higher than in plant-based beef products (4.6 ± 3.6 g vs. 0.8 ± 1.2 g, *p* < 0.0001).
- Protein: For classification Ⅰ, there was no statistical difference between meat-based product and its corresponding meat products except plant-based seafood and seafood. The mean of protein in seafood was nearly 58% higher than that in plant-based seafood (*p* = 0.0013). Analyzing from the angle of classification Ⅱ, almost all the meat-based catalogues had slightly higher protein content than plant-based meat, among which four classes showed statistical significance. The situation was reversed in sausage and its equivalent, where the mean of protein in plant-based sausage was 17.4, nearly 35.9% more than that in sausage (*p* = 0.0031).
- Salt: Salt of both plant-based and meat-based products showed no statistical differences in the categories of plain and breaded meat. Significant differences can be seen in the other four categories where mince showed obviously lower mean of salt than plant-based mince (0.2 ± 0 in mince vs. 1.1 ± 0.9 in plant-based mince, *p* < 0.001), while others showed the opposite outcome.
- Carbohydrate: Plant-based beef and plant-based seafood had similar contents of carbohydrates compared to corresponding meat types, while plant-based pork and plant-based poultry showed 51.1% and 48.5% higher carbohydrates per 100 g than corresponding meat types, respectively (*p* < 0.01). Analyzing from classification Ⅱ, plant-based products (i.e., sausage, plain meat, jerky, and meatball), except plant-based breaded meat and plant-based burger, had significantly higher carbohydrate content than meat (*p* < 0.05) (Table 4).
- Sugar: For classification Ⅰ, the difference was only significant in beef and plant-based beef (6.9 ± 12 vs. 2 ± 2.5, *p* < 0.01). For classification Ⅱ, plant-based jerky and mince had obvious lower sugar than corresponding meat-made products (*p* < 0.01).

### 3.2. Nutritional Quality Assessment

#### 3.2.1. Nutrient Reference Values in Relation to CDRI

The mean NRV and its min–max range of plant-based products according to the CDRI is presented in Table 4. For classification I, the mean NRV of PBMs to energy and fat varied from 8% to 10% and 13% to 19%, respectively. For example, beef analogue provided as much as 13% and 32% for recommended daily intake of fat and saturated fat, respectively. In view of carbohydrates, PBMs contributed a little to daily intake that the mean NRV varies between 3% and 4%. For salt, PBMs demonstrated the feature of high salt, with the mean NRV varies between 18% and 29%. As illustrated by another part of Table 4, from the perspective of classification II, the mean Nutrient Reference Values (NRV) to energy exhibited variability ranging from 8% to 11%, with the highest value observed in plant-based jerky. For fat and saturated fat, the mean NRV of PBMs varied between 11% and 22% and 4% and 20%, respectively, with the example that sausage analogues took up the 22% and 20% of daily fat and saturated fat intake, respectively, which were the highest among all the analogue categories. The mean NRV of PBMs to protein and salt were between 21–35% and 2–8% respectively. From the data, we can see that PB jerky showed the feature of high protein and high salt with the NRV of 35% and 8%. For carbohydrates, the mean NRV of PBMs varied between 2% and 8%.

#### 3.2.2. Evaluation Using Front-of-Pack (FoP) Criteria

According to Table 5, 151 PBMs were classified as three groups. We calculated the percentage of each label, and the result is as follows:

From Table 5, about 90% of PBMs were labeled as medium to high in fat. Over half of the PBMs had zero or low saturated fat with the standard. For salt, 19% and 64% were labeled as zero and low, respectively. PBMs showed the feature of relatively high sugar, as nearly 99% of the PBM products were labeled as medium or high. Referring to the Reduction of Dietary Sodium and Sugar in Hong Kong, over 80% of plant-based meat products met the low-salt standard. While most of the plant meat products belong to medium to high sugar content products, it is difficult to make a definite conclusion on the sugar content of plant meat as there is no clear division between medium and high.

### 3.3. Ingredients Analysis

**Figure 4:**
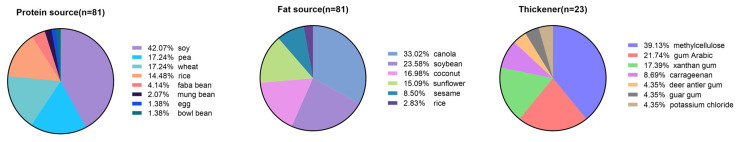
Protein and fat sources and thickeners of plant-based meat analogues (PMBAs) ^1^ on the Hong Kong retail market. ^1^ Total number of products 81. All protein and fat sources listed in the ingredients list, i.e., more than one protein and fat source for some products.

As shown in Figure 4, we investigated the ingredient lists of 81 of 151 plant-based meat products. From the point of view of the main protein source, most manufacturers chose to use soy as the main protein source (42.07%). Wheat and peas were also major sources of protein. In terms of fat source, all products were chosen to use vegetable oils as their source of oil. Of these, most products used canola (rapeseed) oil and soybean oil (33.02% and 23.58%). In addition, there were not many products that declared the use of thickeners on food labels, with methylcellulose being the more mainstream thickener for vegetable meat products.

## 4. Discussion

This cross-sectional study gave an overall profile of PBM products available on the Hong Kong market. The nutrition of plant-based products was assessed through subgroup comparisons with corresponding meat products (two classification methods) using nutrition contents analysis with a nutrition labeling scoring system and local “front of package” labeling standards. On the one hand, our findings presented that plant-based products had a roughly better nutrient quality than meat products. All the contents of macronutrients of PBMs were significantly lower than those of meat excluding carbohydrates after controlling for the categories under two classifications. Through subgroup comparison, despite certain similarities in the content variations between different types of plant-based meat products and their corresponding meat-based counterparts, heterogeneity also prevails, and some differences became insignificant when controlling for product types. On the other hand, PBMs have some limitations and room for progress. According to FoP standards, very few PBMs were attributed as “low sugar” or “low fat”. The features may be ascribed to ultra-processed and processed production [29]. For example, in the analysis of minced meat, the sugar content of animal meat was all zero, which was attributed to the commercially available minced animal meat being primarily raw meat with little addition of sugar. In contrast, PBM, as an ultra-processed food item, carried some additional sugar to enhance the flavor and texture. Overconsumption of sugar, fat, and salt could result in liver and cardiovascular disease [30]. Therefore, manufacturers should reduce sugar and fat to improve the nutritional quality of plant-based products.

### 4.1. Comparison with Other Studies and Criteria

As demonstrated in Table 6, according to existing local products research, PBMs illustrated significantly lower energy density, total fat, saturated fat, and protein, and higher fiber [20,21]. These results were consistent in our study. However, our research lacked the data of fiber content due to the absence of fiber content from the regulation for mandatory nutrients in Hong Kong. In addition, we found salt content of PBMs differs between studies. A study from the United Kingdom [21] presented that PBMs had moderately higher salt than meat products, while a study in Australia suggested no difference [31]. Our study showed the opposite conclusion, that PBMs available in the market of Hong Kong had significantly lower salt content than meat products. Geographical factors and eating patterns may be the reason for the difference. Meanwhile, divergence in the investigation time is another factor in the discrepancy in salt content. As an emerging food product, plant-based meat has seen a very high iteration of product innovation in the industry. Our study was conducted at the end of 2022, one to three years later than the existing literature we compared results with. During this period, manufacturers may have modified and modernized the nutritional content of plant-based meat in order to make it a healthier alternative. Moreover, our analysis showed no different or relatively lower sugar content compared to corresponding meat-made products, which is contrary to the results of the study in Australia [31]. It also indicates the development of PBMs. Nevertheless, according to FoP criteria, few plant-based meat products meet the sugar reduction target, implying a future direction for product innovation.

Focusing on a specific type of vegetable plant-based meat analogue, a great amount of research has been conducted. Our study found plant-based sausages were the only group that had significantly higher average protein (mean per 100 g) than meat products. Contrast to our results, a lower protein content (mean 13.4 g per 100 g) and an opposite conclusion were reported in a study based in Sydney [31]. For plant-based burgers, Alessandrini, Brown, Pombo-Rodrigues, Bhageerutty, He, and MacGregor [21] demonstrated similar fat content (mean 10.3 g per 100 g) while Curtain and Grafenauer [31] reported relatively lower fat content (mean 7.2 g per 100 g), compared to our study (10.6 g per 100 g). We also found that plant-based burgers available on the United Kingdom market [21] contained higher carbohydrates and sugar but lower protein than on the Hong Kong market. The difference could be due to the number of different types of burgers included, e.g., more burgers with bread instead of single burger meat.

A few studies analyzed PBM from the angle of meat type (i.e., beef, seafood, pork, and poultry). For example, Harnack, Mork, Valluri, Weber, Schmitz, Stevenson, and Pettit [22] conducted macronutrient analysis on plant-based beef. Bakhsh et al. [32] explored the textural and physicochemical properties of plant-based beef and pork respectively. Our study discovered plant-based beef, seafood, pork, and poultry showed obvious differences in content of macronutrients. The degree of similarity to meat counterparts varied as well. It is meaningful to figure out the gap and make corresponding improvement in terms of some specific type of PBMs, considering market demand simultaneously.

### 4.2. Healthy Attributes of Plant-Based Meat

#### 4.2.1. Fat Source

**Figure 5:**
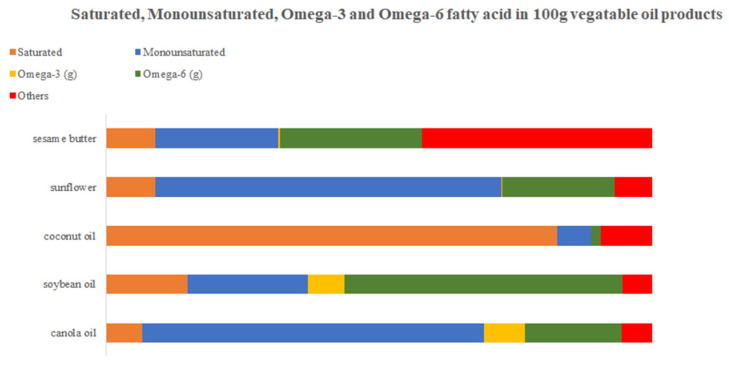
Saturated, Monounsaturated, Omega-3 and Omega-6 fatty acid per 100 g of product [36].

The results of the nutrient analysis showed that most plant-based meats contained less fat and less saturated fat than animal meat products. From the perspective of FoP standards, close to 10% of vegetable meat products were zero or low-fat products. According to our statistics on fat sources in the ingredient list, canola oil, soybean oil, coconut oil, sunflower oil, and sesame oil were the top five vegetable oil sources (Figure 5).

In this study, the ratios of saturated fatty acids, monounsaturated fatty acids, and omega-3 and omega-6 fatty acids per 100 g of the above vegetable oils were calculated using the USDA database (Figure 5). As can be seen from Figure 4, four of the vegetable oil sources, excepting coconut oil, had a relatively low saturated fatty acid profile. Many studies have shown a strong association between excessive intake of saturated fatty acids and cardiovascular disease [33]. According to the dietary guidance of AHA, to achieve a healthy dietary pattern, saturated and trans fats (animal and dairy fats, and partially hydrogenated fat) should be replaced with non-tropical liquid plant oils [34].

Sunflower and canola oil included higher levels of monounsaturated fatty acids. All five vegetable oils were quite low in omega-3 fatty acids; α-linolenic acid (ALA) is the main component of omega-3 fatty acids in plant-based foods. However, foods originating from plant sources do not contain DHA and EPA [35]. EPA and DHA are fundamental to the development and maintenance of the brain, retina, and cell membranes, and have a profitable impact on pregnancy outcomes and the risk of cardiovascular disease (CVD) [35]. ALA can be convertible endogenously to EPA and DHA, but this process is slightly inefficient and is subject to the influence of gender, dietary composition, health status, and age. Therefore, we recommend that people who consume vegetable meat for a long duration should also monitor and intake EPA and DHA foods periodically.

What is worth mentioning as a major surprise is that all five oil sources contain a relatively diverse percentage of fatty acids and four of them have low content of saturated fatty acids.

#### 4.2.2. Protein Source

Table 7 summarizes the protein quality of egg, milk, and some plant protein sources, including soy, wheat, rice, and peas. The digestibility rate and PDCAAS score of soy protein are very close to milk and whey protein. The EEA content of plant proteins is generally inferior to that of animal proteins. Soy, pea, and rice are very comparable to eggs in terms of EAA content. The biological value of plant-derived proteins is dramatically lower than that of animal-derived proteins, and the biological value of soy protein is comparatively high among plant-derived proteins. Compared to other plant-based proteins, soy protein is considered to have a high protein quality.

Our study indicates that soy is the predominant protein source for plant-based meat, with more than 75% of plant-based meat products using soy as a protein source. In more than 15% of products, peas and wheat are also utilized as protein sources. However, in the case where the digestibility and PDCAAS of plant-derived proteins are approaching that of animal proteins, the low EEA content of plant-derived proteins still demands further consideration in design and development of PBM.

#### 4.2.3. Micronutrients

Meat is a source of important micronutrients such as zinc, iron, and vitamin B12 [37,38]. These micronutrients are neither present in plant-derived foods nor are they poorly bioavailable [39]. As a prospective substitute for animal meat, the micronutrient content of plant-based meat and the recommended intakes are subject to further deliberation.

#### 4.2.4. Vitamin B12

Vitamin B12 is exclusively found in meat, which cannot be compensated for with plant-derived provitamins [39]. A recent investigation carried out in the US reported that plant-based minced beef had lower levels of vitamin B12 [22]. An overwhelming proportion of foods derived from plant sources are devoid of vitamin B12. Vitamin B12 deficiency is evident both clinically in the blood and in the nervous system, where cobalamin plays a pivotal role in cell replication and fatty acid metabolism [35,40]. The symptoms and severity of Vitamin B12 deficiency depend on the duration and degree of the deficiency [41]. One of the most common hematologic manifestations of vitamin B12 deficiency is megaloblastic anemia [42]. Vitamin B12 deficiency also has some neurological effects, such as peripheral neuropathy and subacute combined degeneration of the cord and autonomic e.g., bowel/urinary incontinence and erectile dysfunction [43]. Recognizing the indispensable nature of vitamin B12 for health, we also make recommendations in two respects. For consumers who have been consuming PBM as an alternative to animal meat for a long time, especially adults over 75 years old and people with long-term use of metformin or acid-suppressing medications [42], we recommend this population to take a reliable daily supplement of vitamin B12 [35]. For manufacturers who develop PBM, they can fortify their PBM products with vitamin B12 content with reference to the regular vitamin B12 content of animal meat and indicate the content and the recommended intake on the food label.

#### 4.2.5. Iron

Iron (Fe) is one of the essential micronutrients available to humans. Iron deficiency leads to anemia, the most common nutritional disorder. Most people rely on plant-derived foods as their main source of iron, but plants are a poor source of iron in the diet [44]. Plant-derived foods contain non-hemoglobin iron, whereas animal meat contains heme iron. Heme iron is absorbed more efficiently (15–40%) than non-heme iron (1–15%) [45], while non-heme iron is inhibited by phytates, polyphenols, and the proteins in milk and eggs. In the meantime, the absorption of iron from plant-based dietary sources can be ameliorated through the consumption of vitamin C-rich fruits and vegetables [46]. However, in an Australian study, approaching 90% of plant-based meat products were not fortified with iron [47].

Therefore, PBM eaters are reminded of the necessity to be mindful of their dietary iron intake and to replenish regularly with iron-rich foods, particularly for those on diets that feature milk and eggs as the main protein sources.

#### 4.2.6. Zinc

It is predicted from dietary modelling that some substitution of red and processed meat with plant-based alternatives may have a detrimental effect on zinc intake [48]. However, a combination of dietary observations and dietary modelling has also revealed that PBM can be a healthier alternative to animal meat when designed to be fortified with zinc and iron [49]. Studies have demonstrated that the zinc intake and status of vegetarians were lower than that of meat eaters, especially in women [50,51]. Several studies have shown that low concentration of maternal serum zinc in pregnant women has a close link to the incidence of gestational diabetes [52,53]. We consequently recommend that PBM eaters, especially pregnant women, require periodic supplementation with zinc-reinforced foods.

### 4.3. Limitations and Uncertainties

Firstly, this study was not sure to include all plant-based meat products on the market. However, we statistically counted the overwhelming proportion of products available on mainstream sale channels. Secondly, selection bias possibly exists in sampling, as we did not collect all the product data available in the Hong Kong market. Thirdly, although we analyzed PBMs from two classification methods respectively, which control the internal difference of products from different angles, we cannot avoid in-group variation. In addition, this study focused only on nutrients that are required to appear in local food labelling regulations. However, non-obligatory nutrients such as vitamins, minerals, etc. were not counted. We have also assembled several countries or regions on the regulation of food labeling (Table 8). As can be observed from Table 8, the United States has the widest number of nutrients that must be declared, and the European Union is the latest to revise its guidelines. However, apart from the USA, no other country or region has extended vitamins and minerals to nutrients which are mandatory to declare. Our research suggests that given the special characteristics of PBM products, nutrients of plant origin that cannot compensate for animal meat, such as vitamin B12, may be considered for inclusion in the labeling requirements for PBM products. We also suggest that PBM manufacturers add trace elements which are unique to animal meat in the development process to better enhance the nutritional value of PBMs.

### 4.4. Greenhouse Gas Emissions (GHGE)

Food manufacturing is one of the most influential regions of environmental concern in human activity. Greenhouse gas emissions (GHGE) are also an important metric to consider for the plant-based meat industry.

**Figure 6:**
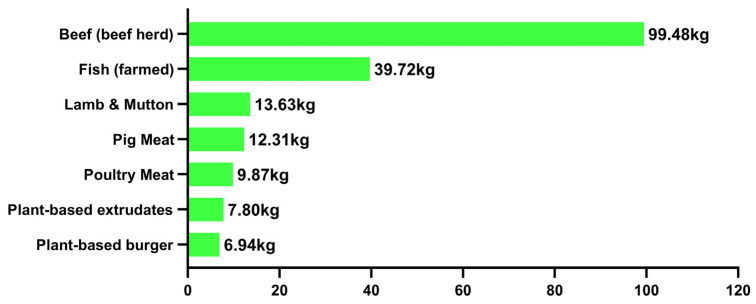
Greenhouse gas emissions per kilogram (CO_2_ eq. kg^−1^) of food products. [61,62,63].

We have also collected GHGE data for selected animal and plant-based meat products as illustrated in Figure 6. Animal meat has dramatically more greenhouse gas emissions than plant-based meat products. Thus, plant-based meat is an earth-friendly alternative.

### 4.5. Future Perspectives

For the authorities and food regulators, there is still a continued emphasis on refining and reinforcing the labelling regulations and management of PBM products, recognizing that PBM is a new type of ultra processed food. We recommend that the FOP requirements for food labelling of PBMs should introduce the indication of dietary fiber and related micronutrient content. Considering that soy as a mainstream protein source for PBM is an allergen, food labelling regulators also must supervise manufacturers to have prominent warnings and declarations of allergenicity on the labels.

For the researchers and manufacturers of PBM, it is essential to take into consideration not only the discrepancies between proteins and oils of plant origin and those of animal origin, but also the level of micronutrients contained in animal meat and their bioavailability.

For nutritionists and food scientists, the subject of “whether plant-based meat is a healthy alternative to animal meat” will also remain a debated topic in the future. However, we propose to track vegetarians who regularly intake PBM, to fortify them with micronutrients according to dietary recommendations, as well as to periodically monitor the health status of this group in a bid to validate the healthfulness of PBM.

For PBM eaters, the long-term consumption of PBM means that some animal-derived micronutrients such as zinc and iron as mentioned in this article may be deficient. This requires regular supplementation with fortified foods with the appropriate nutrients. In addition, we do not endorse the sustainable substitution of vegetable meat for animal meat in specialized groups such as adolescents and women during pregnancy.

## 5. Conclusions

A cross-sectional study was conducted to profile and evaluate PBMs available on the Hong Kong market. Our results demonstrated that PBMs had significantly lower energy density, total fat, saturated fat, protein, and salt than meat products in general. The nutritional quality varied between products. Therefore, progress could be made on specified types of plant-based products. According to FoP criteria of Hong Kong, very few PBMs were labelled as “low sugar” or “low fat”. This is closely related to the attribution of ultra-processed food. Moreover, the nutritional value of PBMs is still limited by protein and oil sources. Therefore, efforts should be made to develop PBMs as healthy options. Meanwhile, the investigation of additives and long-term impacts of eating PBMs is desired to build a more comprehensive understanding.

## 6. Declaration of Authority

The data, criteria, and nutritional evaluation systems presented in this article are not intended for commercial use and are intended for scientific analysis only.

## Figures and Tables

**Table 1:** (**a**)**.** Classification Ⅰ of PBM categories; (**b**). Classification Ⅱ of PBM categories.

Category	Description
(**a**)	
Plant-based pork	Meat-free products appearing to mimic pork. Products featuring ‘pork’ in the product name are included.
Plant-based beef	Meat-free products appearing to mimic beef. Products featuring ‘beef’ in the product name are included.
Plant-based seafood	Meat-free products with seafood-appearing features such as ‘crab’, ’fish’, ’shrimp’ in the product name. The feature ’ocean’ in the product name is included as well.
Plant-based poultry	Meat-free products appearing to mimic poultry, including ‘chicken’, ‘goose’, ‘duck’, etc.
(**b**)	
Plant-based burgers	Meat-free patties, including either ‘burger’, or ‘patty’ in the product name.
Plant-based sausages and luncheon meat	Meat-free products with features either “sausage”, “ham”, “hot dog” or “luncheon meat” in the product name.
Plant-based mince	Meat-free products with features either ‘mince’, ’ground’, or ‘crumble’ in the product name.
Plant-based plain meat	Meat-free products appearing to mimic chicken, beef, goose, seafood, etc. They do not contain gluten and are usually with a little or without sauce.
Plant-based breaded meat	Meat-free products which are breaded, including either ‘fried’ or ‘crispy’ in the product name or products without related key words, but default to be wrapped with bread, such as ‘chicken nugget’, ‘fish finger’, etc.
Plant-based meatball	Meat-free products appear to mimic meatballs. The product name features ‘meatball’, ‘beef ball’ or ‘pork ball’.
Plant-based jerky	Meat-free products feature either ’jerky’, ‘strip’, ‘tip’ or ‘slider’. It is differentiated from plain meat through whether they are covered with much mince/sauce.

**Table 2:** Estimated energy requirement (EER) and recommended nutrient intake (RNI) and upper limit-acceptable macronutrient distribution range (U-AMDR) ^1^ of nutrients ^2^.

	EER
Energy (kcal)	2350
	RNI
Fat (g)	65
Protein (g)	60
Carbohydrates (g)	352
Salt (g)	5.5
	U-AMDR
Saturated fat (g)	26

^1^ Chinese Dietary Reference Intakes (CDRI) does not set the lower limits for AMDR of saturated fat and does not have any recommendation for total sugar intake. ^2^ Values are from the CDRI [26]. Average values for men and women aged 18–50 years with an average level of physical activity.

**Table 3:** Requirements and conditions for nutrient content claims and comparative claims ^1^.

Content	Zero (Not Contained)	Low	Medium to High
Fat	≤0.5 g/100 g	≤3 g/100 g	>3 g/100 g
Saturates	≤0.1 g/100 g	≤1.5 g/100 g	>1.5 g/100 g
Sodium (Salt)	≤5 mg/100 g	≤120 mg/100 g	>120 mg/100 g
Sugar	≤0.5 g/100 g	≤5 g/100 g	>5 g/100 g

^1^ Since only “not contained” and “low content” are regulated in the nutrition labeling regulations, we interpret the range above “low content” as medium to high content.

**Table 4:** Percentage of Nutrient Reference Values (NRV) ^1^ of 100 g of plant-based meat and meat in classification Ⅰ and II referring to the Chinese Dietary Reference Intakes (CDRI) ^2^.

Classifications	Categories	NC Energy	NC Fat	NC Protein	NC Saturated Fat	NC Carbohydrate	NC Salt
Classification I	PB beef	8% (5–18%)	13% (2–31%)	32% (20–67%)	3% (0–17%)	3% (1–8%)	18% (4–33%)
	PB pork	10% (3–32%)	16% (1–37%)	27% (3–103%)	13% (0–68%)	4% (1–14%)	29% (9–76%)
	PB poultry	8% (5–12%)	14% (3–31%)	25% (16–35%)	4% (0–14%)	4% (1–9%)	24% (15–44%)
	PB seafood	8% (1–11%)	19% (0–75%)	17% (0–34%)	7% (0–52%)	3% (1–8%)	24% (11–55%)
Classification II	PB Sausage	10% (7–14%)	22% (10–32%)	29% (17–59%)	20% (3–42%)	3% (1–9%)	27% (16–55%)
	PB breaded meat	8% (6–11%)	14% (2–24%)	22% (4–33%)	4% (0–10%)	4% (1–9%)	22% (15–35%)
	PB burger	9% (6–16%)	16% (8–30%)	21% (8–32%)	14% (0–68%)	4% (1–10%)	18% (15–31%)
	PB jerky	11% (5–16%)	11% (6–18%)	35% (21–56%)	4% (0–7%)	8% (1–14%)	42% (15–76%)
	PB meatball	8% (6–10%)	12% (4–27%)	22% (12–28%)	6% (0–22%)	4% (1–8%)	20% (9–40%)
	PB mince	8% (3–18%)	15% (1–37%)	31% (7–103%)	7% (0–42%)	2% (1–6%)	20% (4–73%)
	PB plain meat	8% (1–12%)	17% (0–75%)	23% (0–44%)	7% (0–52%)	3% (1–7%)	25% (11–55%)

^1^ Data are based on mean (min–max) nutrient content for plant-based meat and meat in classification Ⅰ and II (Appendix A). ^2^ Dietary Reference Intakes for energy, recommended intake for fat, protein, and carbohydrates, and maximum recommended intake for saturated fat and salt (Table 2). Average values for men and women aged 18–50 years with an average level of physical activity.

**Table 5:** Percentage of Nutrient Labelling according to FoP.

Content	Zero	Low	Medium to High
Fat	0.66%	7.95%	91.39%
Saturate fat	15.89%	43.71%	40.40%
Sodium (Salt)	18.54%	63.58%	17.88%
Sugar	0.00%	0.66%	99.34%

**Table 6:** Other studies regarding Comparison of PBM and Meat.

Market	Hong Kong	Australia	Swedish	UK
	Our Study	(Curtain, 2019) [31]	(Bryngelsson, 2022) [20]	(Alessandrini, 2021) [21]
Energy Density	lower	lower	/	lower
Protein	lower	/	/	lower
Total fat	lower	lower	/	lower
Saturated fat	lower	lower	lower	lower
Carbohydrate	higher	higher	/	/
Sugar	lower	higher	/	/
Salt	lower	/	equal	higher
Fiber	/	higher	higher	higher

**Table 7:** Protein quality assessment based on animal and plant protein sources.

Protein Type	Protein Digestibility (%)	PDCAAS (%)	Essential Amino Acid (EAA) Contents (% of Total Protein) *	Biological Value (%)
Animal protein sources				
Egg	98	100	32	100
Milk	96	100	39	91
Whey	100	100	43	104
Plant protein sources				
Soy	95	91	27	74
Pea	99	75	30	65
Rice	87–93	53	28	N/A
Wheat	91	42	22	56–68

* EAA is the sum of His, Ile, Leu, Lys, Met, Phe, Thr, and Val. Trp was not measured. N/A: None.

**Table 8:** Food labeling regulation among different countries or regions.

Country/Region	Nutrients (Mandatory to Declare)	Revised Date	Administrative Organization	Source
Hong Kong Special Administrative Region	Energy density, protein, carbohydrates, total fat, saturated fatty acids, trans fatty acids, sodium, and sugar	1 July 2010	Centre for Food Safety	https://www.cfs.gov.hk/english/programme/programme_nifl/programme_nifl_faq.html [54] Accessed on 23 November 2022
Mainland China	Energy density, protein, fat, carbohydrate, and sodium	7 June 2011	National Health Commission of the PRC	http://www.nhc.gov.cn/wjw/aqbz/201106/a054a6affd0e489da150cf2b51a971a7.shtml [55] Accessed on 18 November 2022
United States of America	Energy, total fat, saturated fat, trans fat, cholesterol, sodium, total carbohydrate, dietary fiber, total sugars, added sugars, protein, and certain vitamins and minerals	January 2020	Food and Drug Administration (FDA)	https://www.fda.gov/food/new-nutrition-facts-label/daily-value-new-nutrition-and-supplement-facts-labels [56] Accessed on 11 November 2022
Japan	Energy, protein, fat, carbohydrates, sodium	October 2020	The Consumer Affairs Agency in Japan (CAA)	https://www.caa.go.jp/policies/policy/food_labeling/ [57] Accessed on 6 November 2022
Australia and New Zealand	Energy, protein, fat, saturated fat, carbohydrate, sugars, sodium	17 August 2020	Food Standards Australia New Zealand (FSANZ)	https://www.foodstandards.gov.au/consumer/labelling/panels/Pages/default.aspx [58] Accessed on 23 November 2022
European Union	Energy, fat, saturates, carbohydrate, sugars, protein, and salt	2 November 2022	European Union	https://europa.eu/youreurope/business/product-requirements/food-labelling/nutrition-declaration/index_en.htm [59] Accessed on 23 November 2022
United Kingdom	Energy, fat, saturates, carbohydrates, sugars, protein, and salt.	3 March 2017	Department of Health and Social Care	https://www.gov.uk/government/publications/nutrition-legislation-information-sources/nutrition-legislation-information-sheet--2 [60] Accessed on 23 November 2022

In evaluation using Front-of-pack (FoP) criteria (Table 3), as the FoP criteria only contains definitions for zero and low content, the Fop criteria lacks a clear breakdown for medium and high content products. We therefore strongly advocate that the relevant authorities should further promote the delineation of FoP standards, to facilitate better assessment of the nutritional profile of products by food manufacturers and consumers.

## Data Availability

No new data were created, and all data are available in the main text or Appendix A.

## Appendix A

**Table A1:** (**a**) Mean energy density (kcal/100 g) and nutrient content (g/100 g) ± SD and range of meat and PBM categories in classification Ⅰ. PB = plant based. (**b**) Mean energy density (kcal/100 g) and nutrient content (g/100 g) ± SD and range of meat and PBM categories in classification Ⅱ. PB = plant based.

Class	N	Energy Density	*p*	Protein	*p*	Total Fat	*p*	Saturated Fat	*p*	Carbohydrate	*p*	Sugar	*p*	Salt	*p*
(**a**)															
Beef	53	242.2 ± 87.3 (118–479)	0.0467	21.7 ± 7 (9–36)	0.2281	11.5 ± 7.6 (3–34.2)	0.2153	4.6 ± 3.6 (1.2–16.9)	<0.0001	10.9 ± 15.9 (0–46.8)	0.5331	6.9 ± 12 (0–35.1)	0.008	1.5 ± 1.2 (0.1–4)	0.0283
PB beef	13	188.8 ± 74.9 (126–420)		19.1 ± 7.7 (11.8–40)		8.7 ± 6 (1–20)		0.8 ± 1.2 (0–4.4)		9 ± 7.6 (2.5–26.8)		2 ± 2.5 (0–9.4)		1 ± 0.4 (0.2–1.8)	
Pork	116	282.7 ± 67.2 (89.3–442)	0.0015	16.3 ± 6 (5.4–39)	0.9935	19.2 ± 8.8 (2.7–43.4)	<0.0001	7.1 ± 3.4 (1.3–15.9)	<0.0001	10.3 ± 12.4 (0–44.9)	0.0089	5.6 ± 9.8 (0–38.1)	0.3921	1.9 ± 0.9 (0.1–5.6)	0.0182
PB pork	78	238.5 ± 107.1 (69–751)		16.2 ± 8.4 (2–62)		10.7 ± 5.5 (0.8–24.3)		3.3 ± 3.5 (0–17.6)		15.3 ± 13.7 (1.9–51)		4.6 ± 6.4 (0–23.5)		1.6 ± 0.9 (0.5–4.2)	
Poultry	65	239.7 ± 67.7 (97–457)	0.0002	15.6 ± 3.8 (9–27.4)	0.3406	15.6 ± 6.4 (2.5–33.8)	<0.0001	4.6 ± 2.9 (0.6–11.9)	<0.0001	9 ± 8.2 (0–58.7)	0.0043	2.4 ± 5 (0–38)	0.8917	1.6 ± 0.5 (0.2–2.6)	0.0014
PB poultry	37	195 ± 46.2 (124–282)		14.9 ± 3.2 (9.3–20.8)		8.8 ± 4.3 (2.2–20.3)		1 ± 0.8 (0–3.7)		13.6 ± 6.7 (1.8–31.3)		2.5 ± 3.2 (0–13.6)		1.3 ± 0.3 (0.8–2.4)	
Seafood	34	234.4 ± 94.5 (36.7–479)	0.0083	16.5 ± 6.5 (4.6–28)	0.0013	12.9 ± 8.7 (0–33.4)	0.8831	3.5 ± 2.7 (0–11.2)	0.0724	10.3 ± 13.5 (0–53.3)	0.7236	2.7 ± 8 (0–39.8)	0.2552	1.4 ± 0.8 (0.1–4)	0.7737
PB seafood	17	177.8 ± 52 (35.2–264)		10.4 ± 4.7 (0.2–20.4)		12.5 ± 10.6 (0–48.5)		1.9 ± 3.1 (0–13.5)		11.3 ± 5.9 (3.8–26.4)		1.1 ± 0.9 (0–3.5)		1.3 ± 0.6 (0.6–3)	
(**b**)															
Sausage	63	271.1 ± 55.2 (89.3–416)	0.0111	12.8 ± 2.3 (5.4–18)	0.0031	22 ± 6.3 (2.7–39.5)	<0.0001	7.9 ± 2.6 (1.8–14)	0.0003	5.5 ± 4 (0–21)	0.0113	2.2 ± 2.2 (0–9.7)	0.1381	2.1 ± 0.6 (1–5)	0.0003
PB Sausage	19	234.4 ± 48.8 (168–323)		17.4 ± 5.7 (9.9–35.5)		14.3 ± 3.8 (6.4–20.6)		5.2 ± 3.1 (0.7–10.8)		9.3 ± 5.6 (1.9–30)		1.3 ± 1.6 (0–5.2)		1.5 ± 0.5 (0.9–3)	
Burger	28	286 ± 67.1 (200–442)	0.0003	15.4 ± 3 (9–21)	0.0045	19 ± 9.4 (6.8–43.4)	0.0002	7.8 ± 3.6 (1.9–15.1)	0.0015	10.3 ± 10.1 (0.1–33)	0.3695	1.9 ± 2.1 (0–6)	0.6729	1.4 ± 0.8 (0.3–4.3)	0.0487
PB Burger	19	210.8 ± 58.9 (150–375)		12.3 ± 4.1 (4.7–19.1)		10.6 ± 4.6 (5.3–19.8)		3.7 ± 4.7 (0–17.6)		12.8 ± 8.3 (3.5–34)		2.2 ± 2.5 (0–9.4)		1 ± 0.2 (0.8–1.7)	
Plain meat	45	209.3 ± 82.1 (36.7–457)	0.1194	17.7 ± 4.5 (5.1–28)	0.0002	12.7 ± 7.8 (0–33.4)	0.2979	3.2 ± 3 (0–11.2)	0.0325	6.1 ± 11.3 (0–58.7)	0.0075	3.1 ± 6.8 (0–38)	0.3645	1.5 ± 0.6 (0.1–4)	0.2793
PB plain meat	37	185.4 ± 55.1 (35.2–282)		13.6 ± 5 (0.2–26.3)		10.8 ± 8.3 (0–48.5)		1.9 ± 2.4 (0–13.5)		11.3 ± 5.1 (2.5–23.6)		2.1 ± 2.2 (0–9.3)		1.4 ± 0.5 (0.6–3)	
Breaded meat	35	259 ± 54.7 (180–437)	<0.0001	15.4 ± 5.2 (8.1–28.4)	0.0955	15.1 ± 5.4 (3.5–33.8)	<0.0001	4.3 ± 1.5 (1.5–8.8)	<0.0001	15 ± 5.9 (3.6–33.8)	0.8995	0.6 ± 1.1 (0–5)	0.0708	1.3 ± 0.4 (0.5–2.1)	0.2901
PB breaded meat	24	197.1 ± 37.1 (136–262.5)		13.2 ± 4.4 (2.6–19.7)		9 ± 4.1 (1–15.8)		1.1 ± 0.7 (0–2.7)		15.3 ± 7.5 (1.8–31.3)		1.8 ± 2.8 (0–13.6)		1.2 ± 0.3 (0.8–1.9)	
Jerky	29	334 ± 39.5 (263–441)	0.0033	28 ± 6.1 (19.6–39)	0.0002	7.6 ± 3.7 (2–15.9)	0.709	2.5 ± 1.1 (1.2–5.3)	<0.0001	38.3 ± 5 (24–46.8)	0.0405	29 ± 6.1 (15.2–39.8)	<0.0001	3 ± 0.8 (1.9–5.6)	0.0173
PB jerky	20	262.6 ± 92 (122–371)		20.9 ± 6.1 (12.7–33.3)		7.2 ± 2.2 (4.2–12)		1 ± 0.6 (0–1.7)		28.8 ± 18.9 (1.9–51)		12 ± 8.6 (0–23.5)		2.3 ± 1.1 (0.8–4.2)	
Meatball	43	239.8 ± 98.7 (118–479)	0.0007	14.3 ± 2.6 (4.6–17.7)	0.2411	16.1 ± 9.8 (3–34.2)	0.0006	6.3 ± 4.4 (1.1–16.9)	<0.0001	5.4 ± 3.3 (1.5–17.9)	0.0168	2.2 ± 1.3 (0–5.7)	0.3927	1.8 ± 0.6 (0.9–3.5)	0.0002
PB meatball	12	177.7 ± 28.9 (132.8–230)		13.2 ± 3.5 (7.1–17)		7.9 ± 5.3 (2.5–17.4)		1.6 ± 2.1 (0–5.8)		13.2 ± 9.6 (3.3–29.2)		3 ± 3.1 (0–8.9)		1.1 ± 0.5 (0.5–2.2)	
Mince	31	231.1 ± 64.4 (121–377)	0.1233	23 ± 4.5 (14.4–31.7)	0.2367	14.8 ± 7.6 (4–31.4)	0.0314	5.6 ± 2.8 (1.3–11.3)	<0.0001	0.1 ± 0.3 (0–1.1)	<0.0001	0 ± 0 (0–0)	0.0054	0.2 ± 0 (0.1–0.3)	0.0003
PB mince	17	194.5 ± 97 (69–420)		18.7 ± 14.2 (4–62)		9.7 ± 7.7 (0.8–24.3)		1.8 ± 3.2 (0–11)		8.1 ± 4.7 (2.3–20)		2.3 ± 3 (0–10.2)		1.1 ± 0.9 (0.2–4)	
